# Consistency Analysis of Genome-Scale Models of Bacterial Metabolism: A *Metamodel* Approach

**DOI:** 10.1371/journal.pone.0143626

**Published:** 2015-12-02

**Authors:** Miguel Ponce-de-Leon, Jorge Calle-Espinosa, Juli Peretó, Francisco Montero

**Affiliations:** 1 Departamento de Bioquímica y Biología Molecular I, Facultad de Ciencias Químicas, Universidad Complutense de Madrid, Ciudad Universitaria, Madrid 28045, Spain; 2 Departament de Bioquímica i Biologia Molecular and Institut Cavanilles de Biodiversitat i Biologia Evolutiva, Universitat de València, C/José Beltrán 2, Paterna 46980, Spain; University of Erlangen-Nuremberg, GERMANY

## Abstract

Genome-scale metabolic models usually contain inconsistencies that manifest as blocked reactions and gap metabolites. With the purpose to detect recurrent inconsistencies in metabolic models, a large-scale analysis was performed using a previously published dataset of 130 genome-scale models. The results showed that a large number of reactions (~22%) are blocked in all the models where they are present. To unravel the nature of such inconsistencies a metamodel was construed by joining the 130 models in a single network. This metamodel was manually curated using the unconnected modules approach, and then, it was used as a reference network to perform a gap-filling on each individual genome-scale model. Finally, a set of 36 models that had not been considered during the construction of the metamodel was used, as a proof of concept, to extend the metamodel with new biochemical information, and to assess its impact on gap-filling results. The analysis performed on the metamodel allowed to conclude: 1) the recurrent inconsistencies found in the models were already present in the metabolic database used during the reconstructions process; 2) the presence of inconsistencies in a metabolic database can be propagated to the reconstructed models; 3) there are reactions not manifested as blocked which are active as a consequence of some classes of artifacts, and; 4) the results of an automatic gap-filling are highly dependent on the consistency and completeness of the metamodel or metabolic database used as the reference network. In conclusion the consistency analysis should be applied to metabolic databases in order to detect and fill gaps as well as to detect and remove artifacts and redundant information.

## Introduction

Metabolic reconstruction is the computational process that aims to elucidate the biochemical network of reactions and metabolites which defines the cell metabolism of a certain organism [1,2]. Since metabolic reconstruction is tightly integrated with genomic information, it can be viewed as a detailed functional annotation of the genome [3,4]. In the first stages of a reconstruction, the genome sequence and its annotation are the main source of information used to infer the biochemical pathways of an organism [5]. Furthermore, each entry annotated as an enzyme coding gene usually contains some identifiers, such as Gene Ontology (GO) terms or Enzyme Commission (EC) numbers, which allow the construction of the gene-protein-reaction rules [6], by mapping one or more coding sequences to one or more reactions, through a protein or protein complex. After this, a metabolic database is used to map the enzymatic activities to instances of biochemical reactions, through their EC numbers [7]. A metabolic database typically describes collections of enzymes, reactions and biochemical pathways, which cover most of the known biochemistry [8,9]. Databases commonly used in metabolic reconstruction include the SEED [10], BiGG [11], KEGG [9] or Metacyc [12], among others.

Although the objective of a metabolic reconstruction may be to create an organism's specific metabolic database, in many cases the final goal is to develop a genome-scale metabolic model (GSM), that is to say, an *in-silico* representation of a metabolic network [13]. A GSM can be used to generate hypotheses about the metabolic capabilities of the network through the computational framework known as constraint-based modeling (CBM), which eventually may be experimentally tested [14,15]. Genome-scale reconstruction has rapidly grown in recent years, as has its range of applications [16,17]. Moreover, CBM can be used to improve model formulation by the detection and resolution of inconsistencies. In this sense the analysis of a GSM can be used to refine the annotation of the genome [18,19] and thus to improve model formulation.

In general, inconsistencies will appear as holes in the structure of the network. These holes might indicate either global or organism specific gaps in biological knowledge. Global gaps are reflected in the existence of metabolites with an unknown biochemical fate [20], as well as the large number of orphan enzymatic activities [21,22]. On the other hand, organism specific gaps are commonly associated with genome annotation errors reflected in the absence of enzymatic activities coded in the genome, or in the inclusion of activities that are not present in the considered metabolism [23].

The resolution of inconsistencies in GSMs is known as network curation. This is a decision- making process where wrongly annotated reactions are removed and candidate reactions are included for the purpose of solving model gaps. The prediction of candidate reactions for filling gaps is referred to as the gap-filling problem [23,24]. Several methods have been proposed for identifying and solving inconsistencies in GSMs in an automatic fashion. Some of these methods rely on the application of optimization techniques [18,24–29], while others focus on a genomic approach to find missing genes [30,31]. In general, these methods require a metabolic database to search for candidate reactions. However, in optimization-based methods the database itself is treated as a large metabolic model where the GSM to be gap-filled is embedded [18,32]. Since metabolic databases include, in general, reactions that span across the tree of life, they can be considered as *global networks* or *metamodels* [33].

The analysis of a *metamodel* through the CBM approach can lead to the detection of structural inconsistencies, as well as coupling relations [34], such as enzyme subsets [35]. Remarkably, the presence of inconsistencies in a metamodel will be propagated to any particular GSM derived from it. As a consequence, the consistency of the metamodel is of critical importance in order to reconstruct a consistent GSM. On the other hand, the information derived from the coupling relations present in a metamodel can facilitate the reconstruction and curation of GSMs. Previous studies have focused on the use of a global network to improve the reconstruction of single organisms [33], although a graph-based approach was there adopted rather than the constraint-based modeling [36].

The aim of the large-scale analysis proposed in this paper is to detect both the recurrent errors in the automatic reconstruction process as well the sources of these inconsistencies, and to assess the impact of the metamodel completeness and consistency in the gap-filling process. To this end, a dataset of 130 GSMs of bacteria reconstructed using the Model SEED pipeline [37] was selected for this study, from which a metamodel was formulated. The main reason for choosing the mentioned dataset relies on the fact that the SEED is a wide extended pipeline for metabolic reconstruction. Additionally, it has been used in several works to perform large scale studies of bacterial metabolism, and to draw important biological conclusions [38–42].

A consistency analysis was performed on the metamodel using the unconnected modules approach [43]. The detected inconsistencies were manually resolved using information from the following metabolic databases: SEED [10], KEGG [9] and Metacyc [12]. The resulting consistent metamodel was used as reference to solve the gap metabolites and blocked reactions in each individual GSM, using a modified version of the *fastcore* algorithm [26]. Moreover, each GSM was also gap-filled using the initial metamodel (*i*.*e*. without manual curation). A comparative analysis was performed between both versions of each GSM to investigate the impact of perform gap-filling using an inconsistent reference network. Finally, a set of models of certain organisms not considered during the construction of the metamodel was used, as a proof of concept, to extend the metamodel with new biochemical information, and asses its impact on the gap-filling results.

### Background

Any biochemical network such as cellular metabolism can be represented by its corresponding stoichiometric matrix ***N***_***mxn***_. This matrix, where rows and columns correspond to metabolites and reactions respectively, represents the structure of the network [44]. Moreover, the set of reactions ***J*** indexes can be partitioned into two disjoint subsets: 1) the set ***J***_***INT***_ of biochemical reactions that take place inside the cell, as well the transport reactions that operate between the cell and the surrounding medium; 2) the set of exchange fluxes ***J***_***EX***_, which are auxiliary variables used to represent the rate at which the metabolites that are allowed to cross the system boundary are consumed/produced [45].

The structural properties of such networks may be assessed through the CBM approach. CBM relies on the use of constraints with the aim of reducing the system's functional states, to those that are physiologically more relevant [14,46]. Two fundamental constraints, and a set of reaction bounds, are used to define the so-called flux space. First, the steady state assumption, imposed over the mass balance equation of each metabolite of the network:

N⋅v=0 (1)

where ***v*** is the vector of reaction fluxes, or flux distribution. Second, the thermodynamic constraints, which ensure that the irreversible reactions take non-negative flux values.

$$0\leq v_{j}\forall j\in Irr$$ (2)

where ***Irr*** is the set of the irreversible reaction indexes. Besides, lower and upper bounds are imposed over each reaction to represent additional constraints, such as the maximum capacity of an enzyme and also to model the surrounding environment of a metabolic system, *i*.*e*. by constraining the exchange fluxes ***J***_***EX***_:

$$\beta_{i}\leq v_{j}\leq \alpha_{i}\forall j\in J$$ (3)

where *β*_*i*_ and *α*_*i*_ correspond to the lower and upper bound of reaction ***i***, respectively.

**Definition:** Any non-trivial vector v feasible with respect to Eqs (1) and (2) is called a flux distribution.

The set of all possible flux distribution spans the so-called flux space:

$$F=\left\{\vec{v}\in R^{n}:N\cdot \vec{v}=0,\beta_{i}\leq v_{j}\leq \alpha_{i}\forall j\in J,\beta_{i}=0\forall i\in Irr\right\}$$ (4)

An important step in the reconstruction of a metabolic model is to check the consistency of the network definition. In general terms, the structural inconsistencies of a metabolic model may manifest as blocked reactions, *i*.*e*. reactions which cannot display a steady-state flux other than zero, and gap metabolites, *i*.*e*. nodes in the network through which there can be no steady state flow [24]. Formally speaking, given a metabolic model with stoichiometric matrix ***N*** and a set of irreversible reactions ***Irr***, structural inconsistencies are defined as follows:

**Definition:** a reaction j is defined as **blocked** if for every possible flux distribution **v** the corresponding flux value **v**_**j**_ = **0**.

**Definition:** a metabolite is defined as a **gap** if all the reactions in which it participates are blocked.

Moreover, the relation between blocked reactions and gap metabolites can be unambiguously established by means of the unconnected modules [43], as following defined:

**Definition:** an **unconnected module** (UM) is defined as a connected bipartite sub-graph of blocked reactions connected through gap metabolites.

**Definition:** a metabolic model with a stoichiometric matrix **N** and a set of irreversible reactions Irr is said to be **flux consistent** if it does not contain blocked reactions nor gap metabolites.

## Materials and Methods

### Computational Tools

Constraint-based analysis was performed using the python-based toolbox COBRApy [47]. LP and MILP problems were solved using the Gurobi Solver [48] accessed through COBRApy. Graph algorithms were accessed through Python-based NetworX library [49]. Graphs were draw using the yEd Graph Editor [50]. Computations were done on a desktop computer with an Intel® Core™ i7 CPU 950 processor, with 23.5 GiB, running under Fedora 17 Linux OS. All the scripts developed for this paper were programmed in Python language [51], and can download from the following repository: https://github.com/migp11/consistency-analysis/. The scripts allow consistency analysis to be performed on any model, computing blocked reactions, gap-metabolites and the set of unconnected modules. Moreover, the code allows gap-filling to performed, based on *fastcore* algorithm.

### Dataset

The Model SEED currently includes 236 publicly available models [10]. A total of 41 models were not available in SBML format, and were therefore not considered. The remaining 195 models comprised: 195 bacteria, 1 archaea (*Methanosarcina barkeri str*. *Fusaro*) and 1 eukaryote (*Ostreococcus tauri*). Since this paper focuses on the analysis of bacterial metabolism, the two models corresponding to the archaea and the eukaryote organisms were excluded from the analysis. A total of 27 of the remaining 193 models corresponded to older versions of some of the models, and therefore they were also excluded from the dataset in order to reduce redundancies.

After these filtering steps, a dataset of 166 bacterial GSMs from a wide taxonomic range was obtained. The 166 selected models included a subset of 130 GSMs which corresponded to the dataset first published by Henry et al., albeit with slight differences which are detailed at the end of this section (see also S1B Table), and included twenty-two models optimized and validated using data from gene essentiality and growth condition experiments [37]. Since these 130 models received particular attention during the reconstruction process, and have also been extensively used to perform large-scale studies of bacterial metabolism [38–42], this subset was chosen as a reference to conduct a consistency analysis and construct the first version of the metamodel. On the other hand, the remaining 36 bacterial models available at the SEED (not previously used in the referred studies, [38–42]) were selected to be used as a proof of concept to assess the impact of extending a metamodel by the adding of new models to a pre-existing one (see S1C Table).

The models were downloaded in SBML format [52] from the SEED (http://www.theseed.org). Moreover, when retrieving the models corresponding to the dataset previously published by Henry et. al [37], new versions of three of the models were found, and they were selected instead of the earlier versions. Additionally, the ID for one of the models (Seed39765.1), corresponding to *T*. *crunogena XCL-2*, was not available; however, by using the species name a model with a different ID was found (Seed317025.3), and this model was used to replace the unavailable model. Finally, the models were grouped according to taxonomic range, using the color schema employed in the Fig 2A in the original publication [37] (see S1A and S1B Table). However, the grouping differed slightly from that of the original publication: 2 GSMs belonging to the *Actinobacteria* (light green) and the *Bacteroidete* (dark blue) groups respectively, had been grouped with the *Gammaproteobacteria* (yellow). Additionally, when the biomass equations of the models were compared, it was found that some models share the same formulation. There are a total of 157 biomass equation and the following organisms share a common equation: *M*. *genitalium* G-37 (Opt243273.1), *M*. *pulmonis* UAB CTIP (Opt272635.1); *S*. *typhimurium* LT2 (Opt99287.1), *E*. *coli* K12 (Opt83333.1), *V*. *cholerae* O1 biovar eltor str. N16961 (Opt243277.1); *S*. *frigidimarina* NCIMB 400 (Seed318167.13), *S*. *halifaxensis* HAW-EB4 (Seed458817.7), *S*. *woodyi* ATCC 51908 (Seed392500.5), *S*. *amazonensis* SB2B (Seed326297.9), *S*. *pealeana* ATCC 700345 (Seed398579.6), *S*. *sediminis* HAW-EB3 (Seed425104.6), *Shewanella* sp. MR-4 (Seed60480.18) (see S3D Table for the details). All the details concerning to the description of the dataset can be found in S1B and S1C Table.

### Constraint-Based Methods

Blocked reactions were computed using the fast consistency check method [26]. Its advantage is that it only needs to solve a small number of linear programs in order to detect blocked reactions. In this paper the algorithm was implemented in python. In all the cases, computation of blocked reaction was performed by relaxing the bounds of the exchange fluxes, allowing the external metabolites to be consumed or produced by the system. The set of gap metabolites and UMs were computed as previously described [43]. The manual curation of UMs was performed with the following set of elementary operations: 1) modifications of the reversibility or direction of one or more reactions; 2) addition of intracellular reactions or transporters; 3) inclusion of exchange fluxes; 4) if there was not enough biochemical information to solve inconsistencies, blocked reactions and gap metabolites were removed from the model.

Flux coupling analysis was computed following the algorithm previously described [34]. Moreover, FCA was performed on a modified version of a model where the constant biomass composition imposed by the stoichiometry of the biomass equation was relaxed in the following manner: first, each metabolite contained in at least one of the biomass equations (*i*.*e*. those metabolites that are a biomass component in at least one GSM) was allowed to be drained from the system in an independent way through demand fluxes; second, each reaction corresponding to a biomass equation was removed. Artificial variables (*i*.*e*. exchange and demand fluxes) were not considered for analysis of the coupling relation.

The *fastcore* algorithm used to perform the gap-filling on the GSM was re-implemented in Python programming language according to [32], and is included in the following repository: https://github.com/migp11/consistency-analysis/. The inputs of the algorithms are a metamodel also referred as a consistent global network, a set of penalties, and an inconsistent model. The output is a consistent version of the model which includes a minimal set of new reactions needed to solve the inconsistencies. The algorithms is an iterative procedure that at each step solves linear programs at each step, in order to identify a set of gap-filling reactions that should be added to the model in order to solve a subset of blocked reactions.

## Results

### Large-scale consistency analysis of 130 GSMs

**Fig 1:**
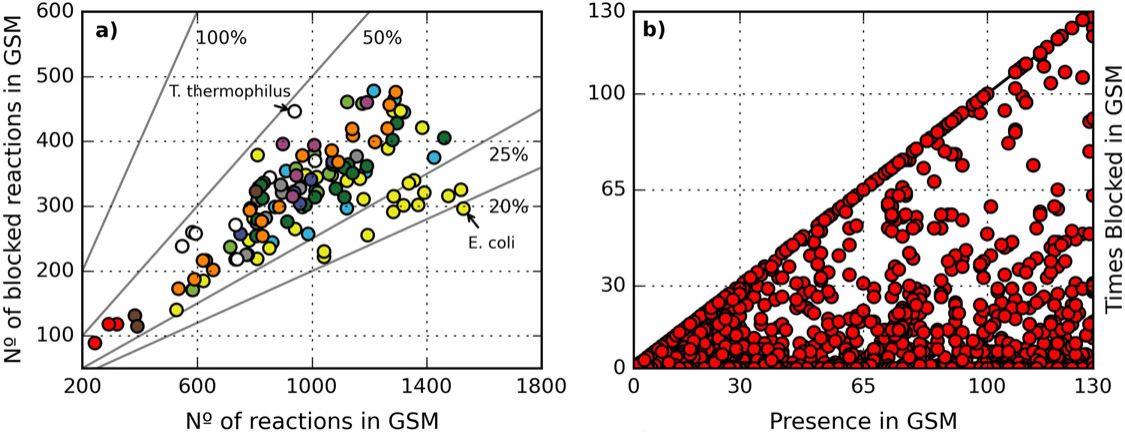
Consistency analysis of 130 GSMs. a) the number of blocked reactions found in each GSM is plotted against the number of total reactions. Grey lines represent iso-probabilities (20%, 25%, and so). Colors of dots correspond to the groups of the Fig 2A previously published [37], and are also included in S1A Table; b) the times a reaction was found in a GSM is plotted against the times it was found blocked. The main diagonal indicates reactions found blocked in all the GSMs where they were present.

Using the dataset previously published by Henry *et* al. [37], composed of 130 GSMs of bacterial metabolisms (see Dataset in Materials and Methods section), a consistency analysis was performed for each model in order to detect: 1) the set of blocked reactions; 2) the set of gap metabolites, and; 3) the set of UMs. As a consequence of this analysis the frequency and distribution of blocked reactions and gap metabolites among the GSMs were determined (see S1A Table). In Fig 1A, the number of blocked reactions versus the total number of reactions for each model is shown. Although there is a high dispersion in the percentage of blocked reactions found across the models, varying between 19–50%, the dispersion is lower than the one represented in the original publication [37]. This is due to the fact that for the present paper the last version of each individual GSM was used (see Data in Material and Methods section). The model with the lowest percentage of blocked reactions (19%) corresponds to the GSM of *Escherichia coli* K12 (Opt83333.1) whereas the highest ~47% was found in *Thermus thermophilus* HB8 (Seed300852.3). Despite this wide range it is still possible to appreciate a trend between the number of blocked reactions and the total number of reactions in a GSM, as previously reported by the authors [37]. This trend suggests that the difficulty of reconstructing a metabolic network increases with the size of the network.

Fig 1B shows the relation between the number of models where a given reaction is present and the number of times the reaction was found blocked (details can be found in S2A Table). Accordingly, 608 reactions (~28%) were never found blocked (points on the x-axis). This subset of reactions includes reactions belonging to well-known pathways and reactions involved in biosynthetic pathways which produce biomass components, such as amino acids or nucleotides (see S2A and S2B Table). Furthermore, a total of 1107 reactions (~50%) were found blocked in some models and active in others, indicating that certain pathways could be correctly reconstructed in some GSMs while in others the reconstruction contained some gaps (see S2A and S2C Table).

**Fig 2:**
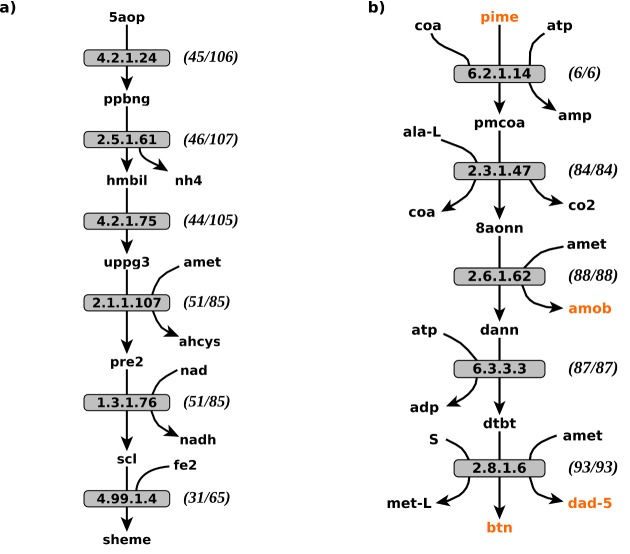
Examples of two metabolic pathways inconsistently reconstructed. a) The siroheme biosynthetic pathway in prokaryotes. b) The biotin biosynthetic pathway from pimelate; yellow colored metabolites indicate root deadends. Numbers in brackets refer to the total number of GSMs in which the reactions were found blocked and present, respectively. Metabolite abbreviations can be found in S3C Table.

The situation described above can be illustrated by the case of the biosynthesis of siroheme (Fig 2A), a prosthetic group present in assimilatory sulfite and nitrite reductases, and found in many prokaryotes. The pathway is mainly composed of three enzyme activities (EC 2.1.1.107, EC 1.3.1.76 and EC 4.99.1.4), and uses uroporphyrinogen-III as a precursor [53]. This pathway is present in 85 GSMs with different degrees of completeness: in 34 GSMs (~40%) it is complete and active; in 28 models (~33%) it is complete but blocked because the end product (siroheme) is not included in the biomass equation, and in 23 models (~27%) there is at least one missing reaction, which causes the remaining ones to be blocked. Due to the fact that uroporphyrinogen-III is also a precursor in at least two other metabolic pathways (*adenosylcobalamin biosynthesis* and *heme biosynthesis)*, the automatic inference of the siroheme biosynthetic pathway shown to be far from trivial.

Finally, 480 reactions (~22%) were found blocked in every model where they are present, as depicted by the points on the main diagonal of Fig 1B. Moreover, a total of 570 metabolites (472 intracellular / 98 extracellular) were always found as gaps. For example, according to the dataset, the synthesis of biotin from pimelate includes five reactions (Fig 2B). Interestingly, none of the models include the synthesis of pimelate, which was an enigma until recently when experimental support for two different routes of synthesis was obtained in *E*. *coli* and *B*. *subtilis [54]*.

The detection of always blocked reactions indicates the existence of recurrent inconsistencies within the reconstruction process (see S2A and S2C Table). These inconsistencies can be artifacts produced during the reconstruction, or the consequence of gaps already present in the metabolic databases. This topic is discussed in the next section.

### Construction and curation of the metamodel

For the purpose to analyzing global inconsistencies, a *metamodel* was constructed by joining the 130 GSMs used in the previous section into a single network. The resulting metamodel, referred as MM130.0, contains a total of 2195 reactions, 1811 metabolites and 370 exchange fluxes (see S3A–S3C Table for details). The metamodel includes a total of 126 different biomass equations because certain GSMs share the same biomass equation (see S3D Table).

A consistency analysis was conducted on MM130.0. The result showed that the metamodel contains 417 blocked reactions and 520 gap metabolites (431 intracellular / 89 extracellular) grouped into 229 UMs (see S1 Fig and S3 Table). As expected, all the reactions classified as *sometimes blocked* (1107), together with the *never blocked* (608) were found to be active in MM130.0. Furthermore, the 417 reactions found blocked in MM130.0 are a subset of the 480 *always blocked* reactions. In the same way, the 520 gap metabolites found in MM130.0 are a subset of the 570 metabolites *always gap*. This indicates that 63 out of 480 *always blocked* reactions and 50 gap metabolites previously classified as *always gap* were “restored” as a consequence of the metamodel construction.

**Fig 3:**
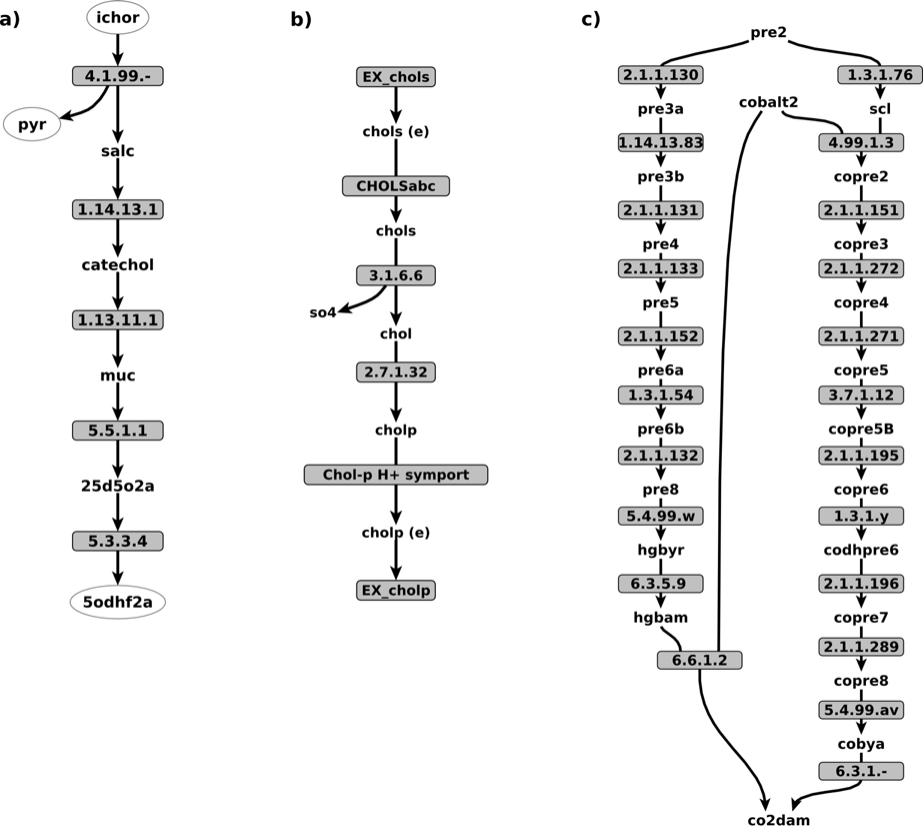
Three examples of pathways unblocked as a consequences of the metamodel construction. In a) a partial representation of the aerobic degradation of catechol is depicted (metabolites in circles were manually added to clarify the diagram). In b) the import of choline sulfate is shown as an example of an in-to-out linear pathway artifact. In c) an example of an artifact is shown, with a set of reactions corresponding to two different variants of the adenosylcobalamin biosynthetic pathway: early cobalt insertion (right side); late cobalt incorporation (left side). Cofactors and currency metabolites were excluded from this representation. Metabolite abbreviations can be found in S3C Table.

In order to understand how this spontaneous connectivity restoring occurred, we mapped these 63 reactions and 50 metabolites onto a bipartite graph, which resulted in 18 connected components (see S2 Fig). Further analysis showed two different scenarios which explain the spontaneous restoring of the connectivity. The first scenario involves the cases of incomplete metabolic pathways in individual GSMs that became complete in the metamodel (Fig 3A). The second scenario corresponds to two classes of artifacts: in-to-out linear pathways and stoichiometric cycles. The in-to-out linear pathways, as the example depicted in Fig 3B shows, are cases where a single metabolite is imported and subsequently excreted, after a few steps. On the other hand, the cycles correspond to *in-silico* flux modes that violate the principle of energy conservation and thus do not have any physical meaning [55,56]. A clear example is depicted in Fig 3C involving two alternative pathways for the biosynthesis of vitamin B12 which, when considered together, form a closed loop. Clearly, both classes of artifacts correspond to a situation with non-physiological meaning, and thus manual curation is needed.

The size distribution of the 229 UMs found in MM130.0 indicates the presence of three different groups. The three biggest UMs included a total of 99 reactions involved in the biosynthesis of membrane lipids and teichoic acids. Interestingly, since lipids and teichoic acids are elongated by iterative reactions, if the corresponding reactions are considered as enzyme activities (*i*.*e*. by considering the set of EC numbers) the three UMs decrease dramatically in size. It is worth noting that the curation of such pathways is particularly difficult. On the other hand, 179 UMs were composed of a single reaction, many of which were unconnected transporters. Within these two extremes, the third group of UMs included a significant number of biological pathways, as well as artifacts. As a consequence of this diversity, the curation of the UMs included in this group usually did not have a trivial solution. Thus, each UM was manually curated according to the procedure described in the Methods section.

As an example, the curation of UM4 (Fig 2B), found in most of the GSMs which involved the transformation of pimelate in biotin [54], is described. This UM contains three deadend metabolites: metabolites: S-adenosyl-4-methylthio-2-oxobutanoate (previously described as a gap [20]), a by-product of 7,8-diaminopelargonic acid synthase (EC 2.6.1.62); 5'-deoxyadenosine (also described as a deadend [57]), which is a by-product of biotin synthase (EC 2.8.1.6); and biotin, the final product of the pathway. For the two first metabolites, and in the absence of information about their further metabolic use, exchange fluxes representing sinks were added. Finally, since biotin is an essential cofactor, its consumption is a consequence of cellular maintenance and growth, and therefore it should be included in the corresponding biomass equations.

**Fig 4:**
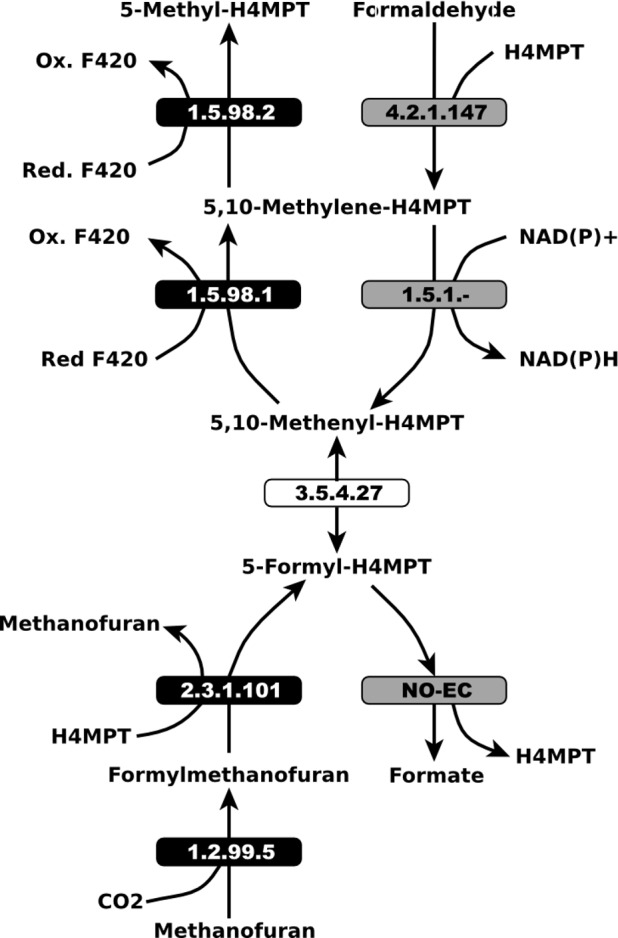
Relation between the pathways: methanogeneis from CO2 and the so-called formaldehyde oxidation V (H4MPT). Black colored reactions were removed from metamodel because they belong exclusively to the methanogenic pathway. The white colored reaction is involved in the metanogenesis but also in the formaldehyde oxidation V (H4MPT) pathway. Grey reactions belonging to the formaldehyde oxidation V (H4MPT) pathway and were manually added to the metamodel to gap-fill the former pathway. Metabolite abbreviations can be found in S3C Table.

Another illustrative example is the curation of UM7, composed of 5 reactions belonging to the methanogenesis, a metabolic process exclusive to members of the domain Archaea (see Fig 4). Strikingly, four of these reactions were found in the GSMs of three proteobacteria: *Burkholderia xenovorans* LB400; *Methylococcus capsulatus* str. Bath; and *Methylobacillus flagellatus* KT. Thus, it is sensible to assume that these reactions were present in these GSMs as a result of errors in the functional annotation of the corresponding bacterial genomes. Interestingly, the three above- mentioned bacterial species code for diverse methylotrophic pathways [58] that in some aspects are analogous and share some enzymes with the methanogenesis pathway [59]. In order to refine the metamodel, reactions with EC numbers 1.2.99.5, 1.5.98.1, 1.5.98.2 and 2.3.1.101 that belong exclusively to the methanogenesis pathway were removed. Furthermore, reactions EC 4.2.1.147, EC 1.5.1.- and reaction formyltransferase/hydrolase (formation of formate from 5-formyl-H4MPT with no assigned EC) were added. These changes, together with EC 3.5.4.27 already present in the metamodel, constitute the so-called formaldehyde oxidation V (H4MPT pathway) as in the Metacyc database. This resulting pathway is more in accordance with the bacterial methylotrophic phenotype.

As a consequence of the curation process, a total of 259 reactions were unblocked. All the reactions added to MM130.0 for solving the different UMs were extracted from the SEED database, except for 22 manually added reactions were based on those found in Metacyc and KEGG (see S1 Fig). Moreover, the reactions and metabolites that were present in non-solvable UMs, referred to as *metablocked* and *metagaps* respectively, were removed from the metamodel. Finally, a set of metabolites considered to be biomass components but not included in the biomass equations, were also found. After the curation process a flux consistent metamodel, named MM130.1, was obtained. Table 1 summarizes the main differences between the initial and final versions of the metamodel. All the details concerning the metamodel curation can be found in S3A–S3E Table (S1 File includes MM130.1 in SBML format).

**Table 1:** Results of the construction and manual curation of the metamodel.

Element	MM130.0 (Initial)	Removed	Added	MM130.1 (Curated)
*Metabolites*	1811	222	77	1666
*† Reactions*	1767	106	230	1891
*Transporters*	428	55	23	396
*Exchanges*	328	52	19	295
* Sinks	—	—	3	3

The first column indicates the elements that define a metabolic model. The second column describes the initial metamodel created by joining all the 130 GSMs. The third and fourth columns indicate the number of elements removed/added respectively as a consequence of the manual curation. The last column describes the final version of the metamodel.

† Biomass equations are not included.

* A sink is a special type of exchange flux, introduced in order to allow a metabolite without a known biochemical fate to leave the system, and thus preventing it from becoming a deadend.

### Flux Coupling Analysis of the Metamodel

The flux coupling analysis (FCA) allows the elucidation of the topological and flux connectivity features of a metabolic network [34]. These features include groups of reactions that operate together in fixed flux proportions under steady-state conditions (*i*.*e*. full coupling sets or reaction subsets [34,35,60]), as well as conditional implication between pairs of reactions (directional coupling). In the context of metabolic reconstruction, we found that the coupling relation of a metamodel allows the immediate resolution of certain gaps during the creation of a specific GSM. For example, given a consistent network ***N*** if a reaction ***j*** is fully or directionally coupled to other reactions, then any subnetwork ***N'*** which includes the reaction ***j*** should also include the reactions required by ***j***, otherwise ***j*** will become blocked. Therefore, if the coupling structure of a metamodel is known, it is possible to detect missing reactions in any model contained on the metamodel.

These results are a consequence of the mathematical properties of the coupling relations, which have previously been studied by Marashi and Bockmayr [61]. The main result proved by the authors states that the coupling relations between any pair of non-blocked reactions will tend to relax (or stay the same) as a network is extended by adding new reactions. Consequently, the coupling relation between any pair of non-blocked reactions will never become stricter (*e*.*g*. directionally coupled → fully coupled) as a consequence of adding reactions. The only case where the coupling between reactions can become stricter occurs when a certain reaction ***i*** is found as blocked in a model, and then becomes active as a result of adding a new reaction ***j***. This special case was considered in the Construction and curation of the metamodel section. On the other hand, when one or more reactions are removed from a consistent network there are different possible outcomes: 1) the couplings between the remaining reactions are not affected; 2) the coupling between some pairs of non-blocked reactions becomes stricter; 3) one or more reactions become blocked. Thus, based on the results commented above, the coupling structure of a metamodel can be used to detect missing reactions in any model that held to be a subnetwork of the metamodel.

FCA was performed on the curated metamodel MM130.1 in order to detect all the coupling relations existing between the reactions in the network. The analysis was conducted on a modified version of model where the constant biomass composition imposed by the stoichiometry of the biomass equation was relaxed (see Methods section). The results showed that the network contains 364 full-coupled sets (FCS) of reactions. Moreover, a total of 1070 (~47%) reactions belong to one of the FCSs present in the model, while the remaining are not involved in fully coupled relations. This indicates that the actual size of the network, in terms of independent reactions, is 1581 instead of 2287. Considering each FCS as a single node, a total of 793 directional coupling relations were also found (see S3 Fig).

The size of the FCSs varies between 2 to 34 reactions with a mean size of ~3 reactions. The biggest FCS is composed of reactions involved in core oligosaccharide biosynthesis, the major component of the outer membrane of Gram-negative bacteria. Other larger FCSs (> 10 reactions) were also involved in the biosynthesis of cell envelope components such as different membrane lipids and peptidoglycan. However, FCSs of a similar size involved in other biological processes were also found, *e*.*g*. the biosynthesis of cofactors and some amino acids.

### Gap-filling of 130 GSMs

The initial metamodel (MM130.0) and its curated version (MM130.1) were used alternatively as the reference network to perform a gap-filling on each individual GSM. In this way, the comparison between models gap-filled with different metamodels can help to assess the impact of the metamodel consistency and completeness on the gap-filling of a GSM.

**Fig 5:**
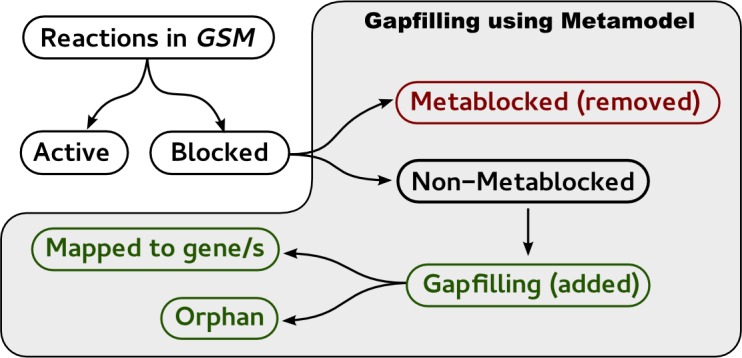
Workflow diagram of the gap-filling process. The set of blocked is classified either as non-metablocked or Metablocked. Metablocked (red) reactions are the ones that couldn't be curated on the metamodel and thus are excluded or removed from the models. Non-metablockeds, are reactions found blocked in at least one GSM but actives in the metamodel and thus can be curated in the GSM. gap-filling reactions (green) are the set of reactions that need to be added to a model in order to unblocked the complete set of blocked non-metablocked. Finally, some of the gap-filling reactions can be associated to one or more genes (mapped) whereas others remain orphans.

The gap-filling was carried out using a slightly modified version of the *fastcore* algorithm to allow the inclusion of a vector of linear weightings, as previously described [32]. Every reaction classified as *metablocked* was removed from individual GSMs before the application of *fastcore*. The number of *metablocked* reactions is 417 and 161 for MM130.0 and MM130.1, respectively. Furthermore, the coupling information obtained from each metamodel version was used to assist the curation process. Finally, the set of weights used in each GSM was individually computed in the pre-processing step. The overall curation process was divided into 5 steps (see Fig 5), as described below.

1. **Pre-processing step:** all *metablocked* reactions present in the GSM are removed.
2. **FCA completion:** for each blocked reaction in the GSM, the set of fully coupled and directionally coupled reactions detected in the metamodel are selected. The selected reactions not already present in the GSM are included before applying *fastcore*.
3. **Generate penalties:** the set of penalties for all the reactions not included in the GSM is constructed as follows: spontaneous reactions, sinks, and exchanges fluxes are weighted to 0. The reactions that have an associated EC-number which matches the one associated to a reaction already present in the model are also weighted to 0. All the remaining reactions are assigned the same penalty, namely a value of 10, as previously described [32].
4. **Gap-filling step:** *fastcore* algorithm is run using a metamodel as the global network reference and the set of penalties generated in step 2.
5. **Post-processing step:** the EC numbers of the gap-filling reactions are compared to those present in the GSM and, if there is a match, the gene-protein-reaction association is transferred to the added reaction.

**Fig 6:**
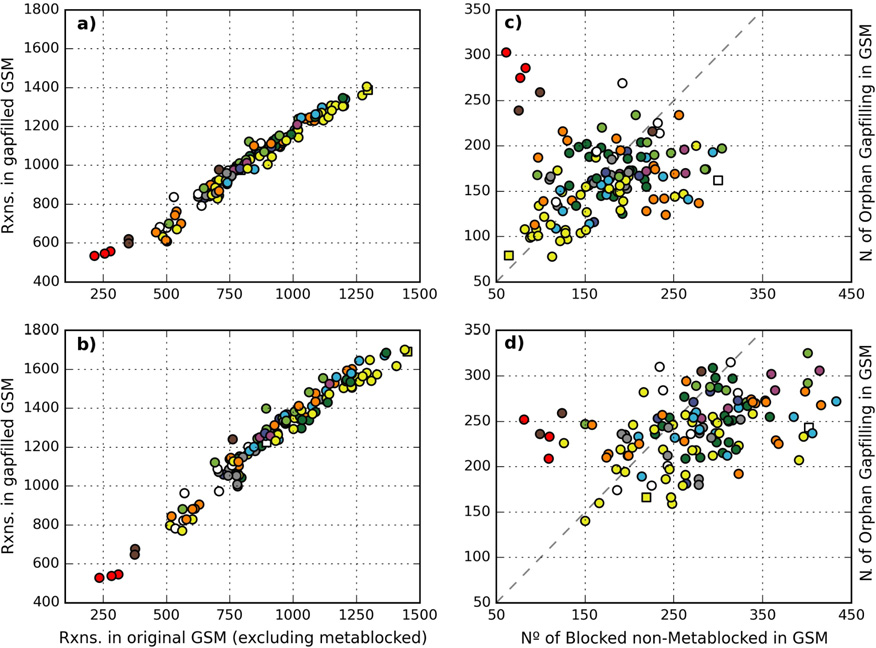
Comparative results of the gap-filling on the 130 GSMs using MM130.0 and MM130.1. In a) and b) the number of reactions in the curated GSM against the number of reactions in the original GSM (not considering metablocked reactions), using MM130.0 and MM130.1 respectively. In c) and d) the number of orphan gap-filling reactions added to each GSM plotted against the total number of curated reactions. Dotted line represents the function y = x. Yellow and white squares correspond to *E*. *coli* and *T*. *Thermophilus*, respectively.

The described procedure was applied to each of the 130 GSMs present in the dataset using alternatively MM130.0 and MM130.1, which resulted in two consistent versions of each GSM. The results showed that in both scenarios the size of the GSMs tended to increase significantly after the gap-filling. As expected, the size of the gap-filled model is, in most of the cases, higher when MM130.1 was used as the reference (Fig 6A and 6B). However, certain models do not seem to be affected by the metamodel used in the curation. This happens for the cases of M. *genitalium* G-37, M. *pulmonis*, OY. *phytoplasma* (red dots), T. *pallidum* and B. *burgdorferi* B31 (brown dots). These five organisms are known pathogens and possess the smallest genomes of the dataset (see S1A Table). Furthermore, the relation between the size of the gap-filled GSMs and their original versions follows a linear relation. The same trend is also found if metablocked reactions are considered in the size of the original GSM (see S4 Fig).

As shown in Fig 6C and 6D, the ratio between the totals of gap-filling orphan reactions and curated reactions was, for most of the models, lower than 1. In such cases this indicates that, on average, solving a blocked reaction requires the addition of a new orphan reaction. Interestingly, *T*. *thermophilus*, the GSM with the largest percentage of blocked reactions, required a relatively small number of gap-filling reactions in order to become flux consistent. However, in several cases the number of orphan gap-filling reactions significantly exceeds the number of blocked reactions that are curated. This suggests that in these cases many blocked reactions can be artifacts, and thus should be pruned rather than gap-filled. This is quite clear for the cases of the five pathogenic organisms with reduced genome mentioned above.

When the reaction content between each pair of curated GSMs was compared, it was found that 121 of the GSMs curated with MM130.1 include at least 90% of the reactions of the version curated with MM130.0. This indicates that, for most of the cases, the consistent GSM obtained when MM130.0 was used as a reference is almost completely included in the consistent version obtained when MM130.1 was used. However, in five models the percentage of inclusion was lower than 80%. These models, which correspond to the five outliers mentioned above, have almost the same number of reactions in both gap-filled versions, and significantly differ when the complete set of reactions is compared.

The manually curated pathways of MM130.1 were correctly gap-filled in the models containing some of the pathways. For instance, biotin biosynthesis was correctly gap-filled in 97 GSMs. Similar results were found for the cases of the siroheme biosynthesis, as well as vitamin B12. Nevertheless, none of these pathways could be gap-filled when using MM130.0, as expected. Moreover, when using MM130.0 as a reference network, and with respect to the GSMs that included at least one reaction which belongs to either of the two alternative pathways for vitamin B12 biosynthesis (see Fig 3), it was found that the gap-filling solution resulted in the inclusion of a futile cycle. This indicates that the presence of such cycles may generate artifacts in GSMs when applying automatic gap-filling algorithms.

### Extending a metamodel with new biochemical information

Metabolic databases such as the SEED or BioCyc are constantly being updated by the reconstruction of new metabolic models, as well as the addition of new biochemical information (*e*.*g*. MetaCyc). New organisms not considered during the construction of MM130.1 may contain reactions and compounds, or even previously unconsidered new metabolic pathways. Consequently, a metamodel will eventually need to be extended or updated. Thus, in order to assess how extending a metamodel affects the performance of gap-filling, 36 GSMs which had not been considered in the construction of MM130.1 were used to extend the metamodel (see Material and Methods section and S1C Table).

The extension process was performed in the following way: each new model was inspected in order to find reactions and compounds that were not present in MM130.1. A total of 243 new metabolites and 331 new reactions were found, and used to extend the metamodel. The first version of the extended metamodel, named MM166.0, included a total of 1909 metabolites and 3047 reactions. A consistency analysis performed on MM166.0 showed that 143 out of 331 new reactions were blocked. Moreover, 119 reactions in the set of blocked reactions were found to have been previously classified as metablocked, and were therefore removed from the metamodel (see the Construction and Curation of the Metamodel section and S3A Table). The remaining 24 blocked reactions were mapped into unconnected modules and manually curated by the addition of twelve new reactions and two exchange fluxes. After this, a consistent version of the extended metamodel, named MM166.1, was obtained. Table 2 summarizes the results of the extension process (S1 File includes MM166.1 in SBML format).

**Table 2:** Results of the metamodel extension with 36 new models.

Element	MM130.1	New added	MM166.1 (Curated)
*Metabolites*	1666	113	1779
*† Reactions*	1891	141	2032
*Transporters*	396	55	451
*Exchanges*	295	30	325

† Biomass equations are not included.

In order to assess the impact of the metamodel extension on the gap-filling of individual models, the process described in the previous section (Gap-filling of 130 GSMs) was repeated, but using MM166.1 as the reference network instead. Moreover, the gap-filling procedure was also applied to the additional 36 GSMs using the two metamodels (MM130.1 and MM166.1) as the reference networks. As a result two consistent versions of each of the 166 modes were obtained. In order to compare the two curated versions of each GSM two factors were considered: 1) the efficacy of the gap-filling algorithm (number of curated reactions *per* added orphan reaction); and 2) the differences between both versions with respect to the gap-filled reactions.

The mean efficacies for the models curated with MM130.1 and MM166.0 were ~0.87 and ~0.93, respectively for the 130 models, and 0.90 and 0.99 for the 36 additional models (see S4 Table). The increase in the gap-filling efficacy when the expanded metamodel was used can be explained by the fact that *fastcore* finds a minimal consistent model by minimizing the number of reactions which should be added to the model in order to solve the set of inconsistent reactions. Therefore, if a bigger metamodel which includes new pathway branches and reactions is used, an improvement in efficacy (as previously defined) is clearly expected. However, this factor does not provide information about the degree of similarity between the gap-filling solutions obtained for each metamodel. Thus, the two versions of each curated model were compared to quantify the degree of similarity between them, in terms of the reactions they contain, using the Jaccard index. The Jaccard index, defined as the total number of elements in the intersection over the number of elements within the union of two sets, was calculated for each pair of models considering only the subsets of gap-filling reactions, *i*.*e*. the reactions added to solve the inconsistent ones.

The mean value of the Jaccard index of the 130 models was ~0.71, and the same mean value was obtained for 36 models, which means that on average each pair of curated versions shares, approximately 85% of the new reactions (see S4 Table). Surprisingly, no significant differences were found between the index calculated for the dataset of the 130 models, and the index for the dataset of the 36 models. This suggests that most of the biochemical information needed to curate the models was already contained in MM130.1 and that the additional 196 reactions added during the extension were probably concentrated in a few models. However, since most of these new reactions were already consistent in the individual models, they are not expected to affect the gap-filling results.

## Discussion

### Finding structural inconsistencies in GSMs

The consistency analysis performed on a dataset of 130 GSMs of different bacteria showed that most of the models have at least 25% of their reactions blocked. The results also showed that the reconstruction of a non-model organism tends to become easier when the GSM of a phylogenetically closer organism exists, as previously discussed [16]. In the analyzed dataset, this is reflected by the fact that the models with less than 25% of their reactions blocked belong to the class of *gammaproteobacteria*, which includes *E*. *coli*.

As expected, the model with the lowest percentage of blocked reactions was the one corresponding to *E*. *coli* K12 (Opt83333.1) with a value of ~19%. The same measure was also evaluated for the last version of the manually reconstructed GSM of *E*. *coli* K12, named *iJO1366 [57]*, and the result showed that 10% of its reaction were blocked. The difference found was expected, since *iJO1366* has been extensively manually curated, whereas Opt83333.1 was automatically reconstructed. On the other hand, *T*. *thermophilus* (Seed300852.3) was shown to be the model with the highest percentage of blocked reactions (~47%). As in the case of *E*. *coli*, this measure was compared against the value obtained for a recently published GSM of *T*. *thermophilus*, named *iTT548* [62], where ~27% of its reactions were found blocked. This value, although high, shows that manual curation dramatically improves the consistency of a GSM. Furthermore, the use of additional experimental data will also help to improve the consistency and completeness of a GSM.

### Gap-filling through metamodel analysis

A comparative analysis surprisingly showed that 480 reactions (~22%) are blocked in each GSM where they are present (*i*.*e*. “always blocked”). This showed a remarkable number of network inconsistencies at the level of the metabolic database used as a reference during the reconstruction process. When these reactions were grouped by subsystem, 25% were found to be involved in the biosynthesis of cell wall components as well as membrane lipids (see S2D Table). This showed that the automatic inferences of such metabolic pathways is particularly hard because of its iterative nature, along with the diversity of cell envelope components, and the absence of experimental information about cell membranes for most of the organisms analyzed. Furthermore, pathways for the biosynthesis of important cofactors, such as siroheme, biotin and vitamin B12, were also found to be always blocked. In some cases the pathway was incomplete whereas in other cases it was complete but blocked. For example, the curation of the biotin biosynthesis is an illustrative example of how models must evolve in parallel to biochemical knowledge (see Construction and curation of the metamodel in result section).

The detection of recurrent inconsistencies motivated a global consistency analysis, *i*.*e*. the analysis of the global metabolic network. In the present paper a metamodel, named MM130.0, including 130 GSMs was reconstructed using the SEED pipeline. The consistency analysis and the visual inspection of the set of UMs founded in MM130.0 allowed: first, the detection of reactions wrongly included during the reconstruction of the individual GSMs (*e*.*g*. some activities belonging to methanogenesis); second, the detection and resolution of incomplete pathways by the addition of the corresponding missing reactions, *e*.*g*. the pimelic acid biosynthesis (*i*.*e*. the first stage of biotin biosynthesis); third, the detection and resolution of global deadend metabolites such as the cases of S-adenosyl-4-methylthio-2-oxobutanoate and of some biomass components not previously included in the biomass reaction of any GSM. The curated metamodel MM130.1 can be used as a reference network for the curation of any model generated using the SEED. In this way, different algorithms and weights can be tested for the curation of a particular model. Additionally, the metamodel presented here can be modified or extended to adjust to particular situation such as the case of organisms with highly reduced genomes.

### Impact of metamodel consistency on automatic gap-filling

Many gap-filling algorithms have been developed by focusing on the structure of the objective function. However, the possible impact of the reference network consistency, which affects the gap-filling process, has received little or no attention. Therefore, a gap-filling was performed on the whole dataset to evaluate the impact of the metamodel completeness and consistency, using alternatively MM130.0 and its curated version MM130.1. When the reaction content was compared within each pair of curated GSMs, in 125 of the cases the version gap-filled with MM130.1 contained 85% or more of the reactions present in the version gap-filled with MM130.0. Strikingly, in five models the percentage of inclusion varies between 65–80%, which indicates a remarkable difference between the two versions. Thus, it can be concluded that in some cases the result of an automatic gap-filling can be highly dependent on the metamodel used as the reference. In particular, the divergence was found to be greater for the smaller models. Since, these organisms correspond to obligate parasite and symbionts, their metabolisms tend to lack many biosynthetic capabilities and thus, they should import a great number of metabolites from the surrounding environment. Furthermore, the possibility of a metabolic complementation between parasites or symbionts and their hosts may result in the exchange of metabolites not commonly included in growth media. Thus solving such situation requires a manual inspection of individual UMs to evaluate the possibility of adding unknown transporters and the corresponding exchanges fluxes [43]. As a consequence, the metabolic reconstruction of such organisms is more susceptible of gap-filling artifacts

Finally, the gap-filling results showed that in many cases the number of orphan gap-filling reactions included to solve gaps significantly exceeds the number of gaps. This result is independent of the metamodel version used, and suggests that in many situations blocked reactions can be artifacts and should be removed from the model instead of adding new reactions to solve what at first looked like a gap. For example, the initial GSM of *M*. *genitalium* G-37 (Opt243273.1) contained 292 reactions, of which 118 were blocked, whereas both gap-filled versions included more than 500 reactions. The manually reconstructed model of *M*. *genitalium* G-37 *iPS189* [28] contains 265 reactions, of which 90 are orphans, which indicates that gap-filled version of Opt243273.1 significantly overestimates the number of reactions. Such kinds of discrepancies clearly indicate that, in this case, most of the blocked reactions should be pruned rather than gap-filled. This result showed that the curation of a metamodel is necessary, but not sufficient condition, in order to perform automatic model curation.

The extension of MM130.1 to MM166.1 by the addition of 36 GSMs allowed us to show how a metamodel can be updated to include new biochemical information. In this particular case the inclusion of 36 additional models provided only 141 reactions and 55 and transporters, and most of these reactions belong to a single organism (*P*. *difficile*). Nevertheless, this new information was enough to affect the outcome of the gap-filling process, as has been shown (see section Extending a metamodel with new biochemical information). Moreover, the increase in *fastcore'*s efficacy when the expanded metamodel was used was expected, taking into account the fact that *fastcore* finds a minimal consistent model by minimizing the number of reactions added to solve inconsistent reactions. Consequently, if new pathway branches are available due to the utilization of a bigger metamodel, an improvement in the achieved minimum is possible. However, this minimization criteria may lead to artifacts such as chimerical pathways composed of reactions of organisms not related to the one being analyzed. Taking everything into account, it is worth to note that the automatic reconstruction and curation of metabolic models should consider the taxonomic distribution of the reactions, so that the weights assigned to them can be properly adjusted when formulating the gap-filling problem, as has been discussed by other authors [29,63].

The reconstruction of a GSM depends, to a great extent, on the quality of the genome annotation. Thus, errors in the annotation will lead to the presence of inconsistencies in the model formulation. In this context, in principle there are two different types of annotation error: 1) the absence of certain functional annotations, *e*.*g*. an enzyme coding gene not annotated as such; 2) the incorrect assignment of an enzymatic activity to certain coding sequences. The presence of type 1 and 2 errors will produce the absence, or the wrong inclusion, of some reactions in the GSM, respectively. Thus, gaps in a GSM caused by distinct types of errors will require a differential treatment. In the case of type 1, missing reactions should be gap-filled. However, in the case of type 2 errors, the wrong added reactions should be removed. Since both types of errors will manifest as blocked reactions and gap metabolites, we argue that the problem resides in how to differentiate, in an automatic fashion, between the blocked reactions that have to be gap-filled (in order to restore their connectivity) and the erroneous reactions that should be removed. As shown in this paper the distinction between these types of errors is far from trivial, and not considering them will result in the overestimation of the reaction content of an organism.

Although there have been considerable advances in the automatic reconstruction of GSMs, the outputted models still contain many structural inconsistencies, as the results presented have shown. Furthermore, the detection of recurrent inconsistencies manifested as metablocked reactions indicates that gaps may already be present in the metamodel or the metabolic database used to perform the reconstructions. As a consequence, we argue that consistency analysis, such as the one presented in this paper, should be applied to metabolic databases in order to detect and fill gaps as well as to remove artifacts and redundant information.

## Supporting Information

S1 Fig Curation of the unconnected modules of MM130.0. The diagram represents the set of unconnected modules obtained for MM130.0, together with other artifacts detected, and set of reactions added during the curation process. White nodes correspond to blocked reaction solved; green nodes correspond to the reactions added during the curation; red nodes indicate the set reaction that were removed from the metamodel (metablocked).
(SVG)

S2 Fig Blocked and gap metabolites “restored” as a consequence of the metamodel construction. The diagram depicted the sub-graph corresponding to the subset of reaction find as always blocked in the dataset of 130 GSMs, which becomes active as a consequence of the construction of the MM130. In a) the sub-graphs the representing cycles which correspond to *in-silico* flux modes that violate the principle of energy conservation. In b) the cases of in-to-out linear pathways are shown (see section “Construction and curation of the metamodel” in the main text). In c) the cases of incomplete metabolic pathways in individual GSMs that became complete in the metamodel are depicted.
(PNG)

S3 Fig Flux Coupling Graph corresponding to MM130.1 with uncoupled biomass reactions. The diagram represents the flux coupling graph associated to MM130.1 as previously described. Grey and red nodes represent single reactions and full coupled sets of reactions respectively. Directed arrows indicate the presence of directional coupling between the pair of nodes.
(PNG)

S4 Fig Comparative results of the gap-filling on the 130 GSMs using MM130.0 and MM130.1. In a) and b) the number of reactions in the curated GSM against the number of reactions in the original GSM are shown (including metablocked reactions), using MM130.0 and MM130.1 respectively.
(PNG)

S1 File Metamodels in SBML format. Compressed ZIP file including both curated metamodels (MM130.1 and MM166.1) in SBML format.
(ZIP)

S1 Table Description of the dataset and the main consistency analysis results. The table contains two sections. In A) the 130 GSMs used in the present paper are listed, including a detailed description of the models as well as the main results of the consistency. In B) the mapping between the version of the models used herein and those used in the original publication [37]. In C) the description of the 36 addition bacterial models used to extend the metamodel.
(XLSX)

S2 Table Classification of all the reactions according to their block-presence frequency. The table contains five sections. In A) the set of 2195 reactions included in at least one GSM. Each row corresponds to a reaction and includes the reaction's attributes together with the number of models which include the reaction, the number of models where it is blocked and the calculated blocked frequency. Sections B), C) and D) presents the reactions (grouped by subsystems) classified as “never blocked”, “sometimes blocked” and “always blocked”, respectively.
(XLSX)

S3 Table Description of the metamodel MM130 and its curation process. The table contains five sections. Section A) includes the complete set of reactions and their description. Additionally, two columns were added to indicate if the reaction was added or removed during the manual curation, and a third column to indicate the unconnected module ID associated to those reaction first found blocked. In a similar fashion, sections B) and C) described the set of exchanges fluxes and metabolites of the metamodel respectively. In D) the 157 biomass equations are presented, indicating the models which have that biomass composition. In E) the list of new biomass compounds found are shown. In F) and G) the new 226 reaction and 113 metabolites, respectively, found in the set of 36 GSM that was used for the metamodel extension.
(XLSX)

S4 Table Comparative results of the gap-filling performed to the 130 GSMs and 36 GSMs, using alternatively MM130.0, MM130.1 and MM166.1 as the reference network. The table contains three sections. The results of the gap-filling performed using MM130.0 and MM130.1 are depicted in sections A) and B) respectively. In section C) the results of the comparative analysis performed on each pair of gap-filled GSM are shown. In D) the results of the gap-filling performed on the dataset of 130 GSMs using MM166.1 are shown. In E) the results of the gap-filling performed on the dataset of 36 GSMs using MM130.1 MM166.1 are shown. In F) the comparative analysis of the gap-filling results obtained using MM130.1 and MM166.1 are shown.
(XLSX)
