# Supplementary figures and images for: Consistency Analysis of Genome-Scale Models of Bacterial Metabolism: A Metamodel Approach
Source: PLoS One. 2015 Dec 2;10(12):e0143626. doi: 10.1371/journal.pone.0143626 (PMC4668087; doi:10.1371/journal.pone.0143626)

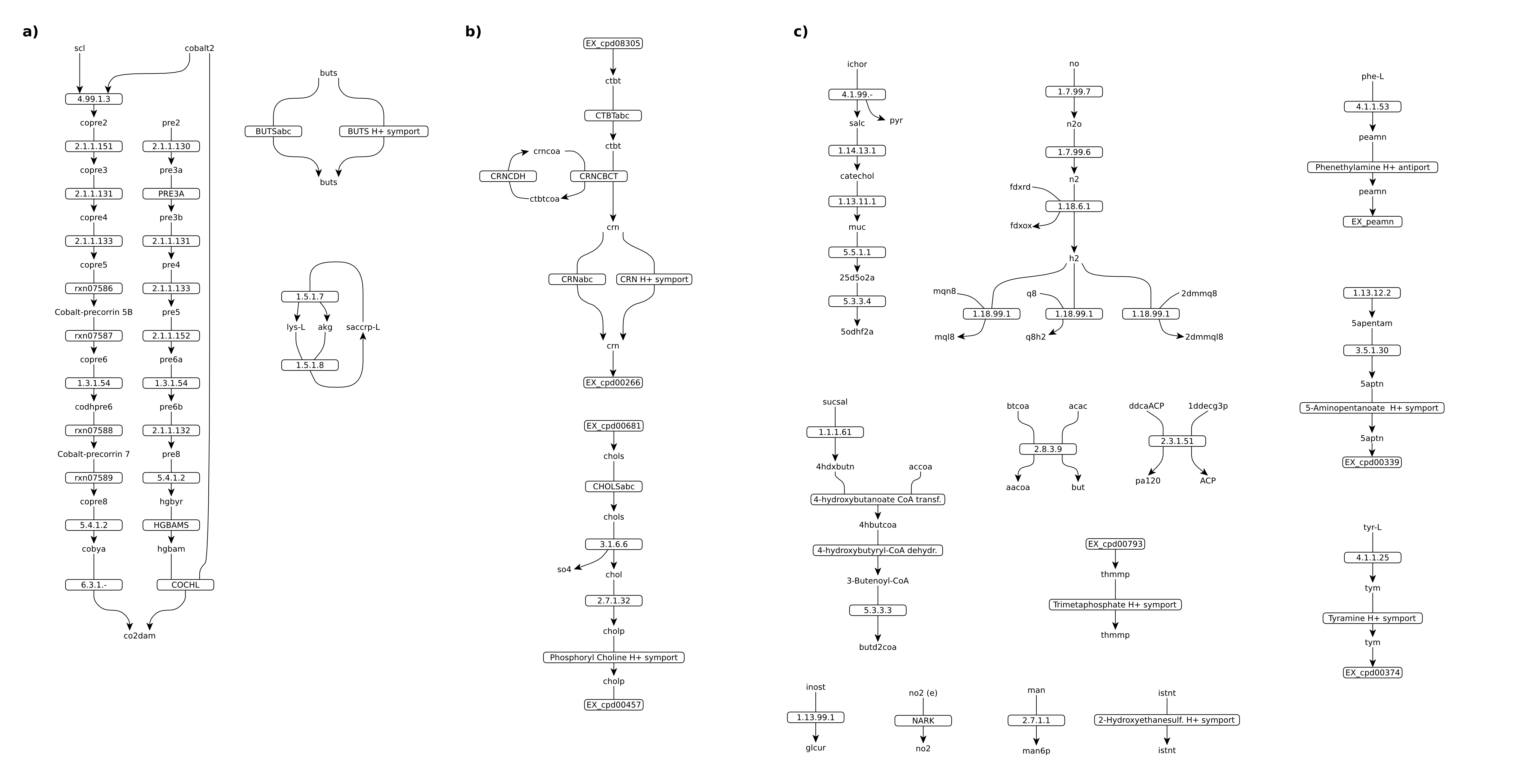

Supplement: S2 Fig — The diagram depicted the sub-graph corresponding to the subset of reaction find as always blocked in the dataset of 130 GSMs, which becomes active as a consequence of the construction of the MM130. In a) the sub-graphs the representing cycles which correspond to in-silico flux modes that violate the principle of energy conservation. In b) the cases of in-to-out linear pathways are shown (see section “Construction and curation of the metamodel” in the main text). In c) the cases of incomplete metabolic pathways in individual GSMs that became complete in the metamodel are depicted. (PNG) [file pone.0143626.s002.png]

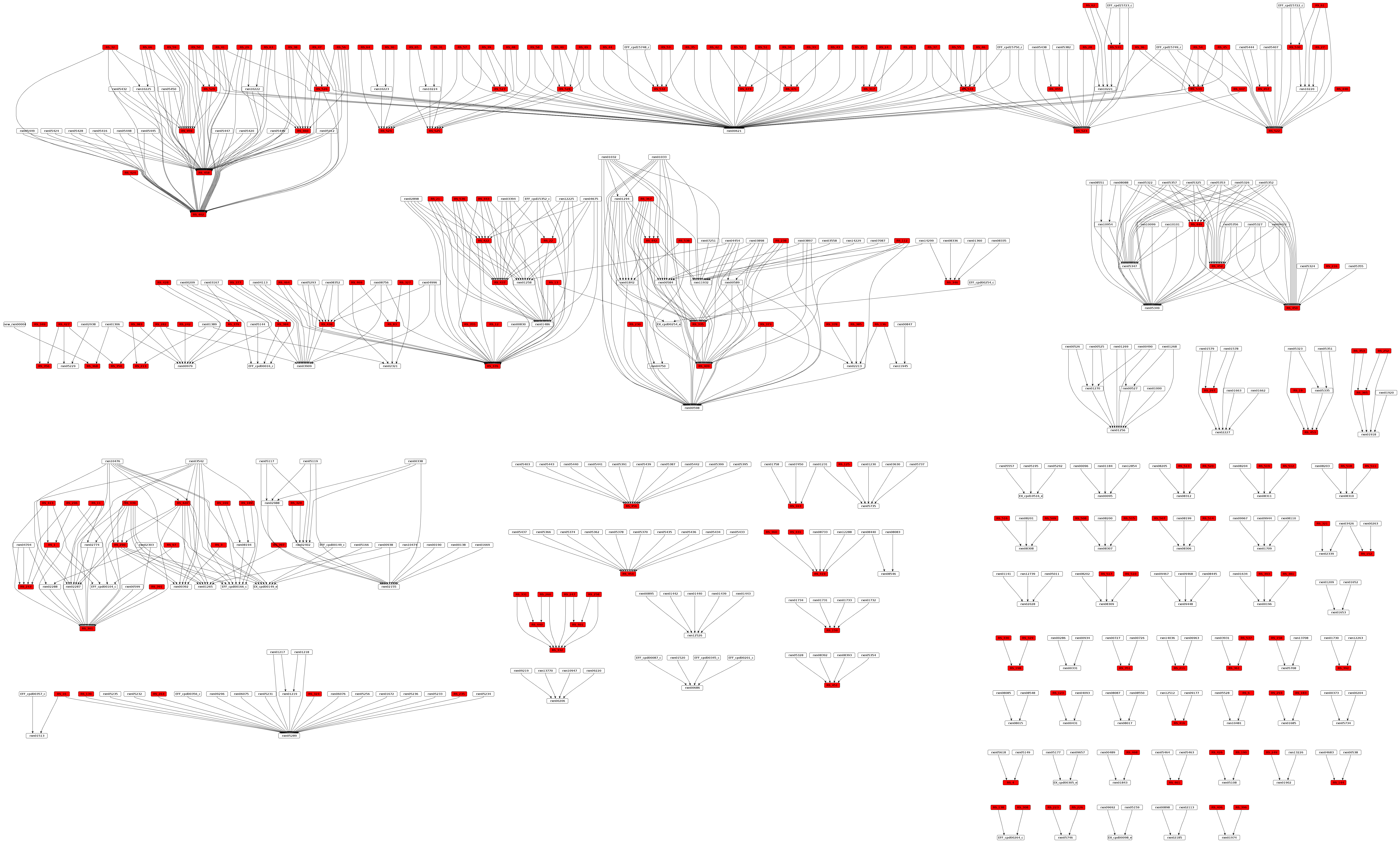

Supplement: S3 Fig — The diagram represents the flux coupling graph associated to MM130.1 as previously described. Grey and red nodes represent single reactions and full coupled sets of reactions respectively. Directed arrows indicate the presence of directional coupling between the pair of nodes. (PNG) [file pone.0143626.s003.png]

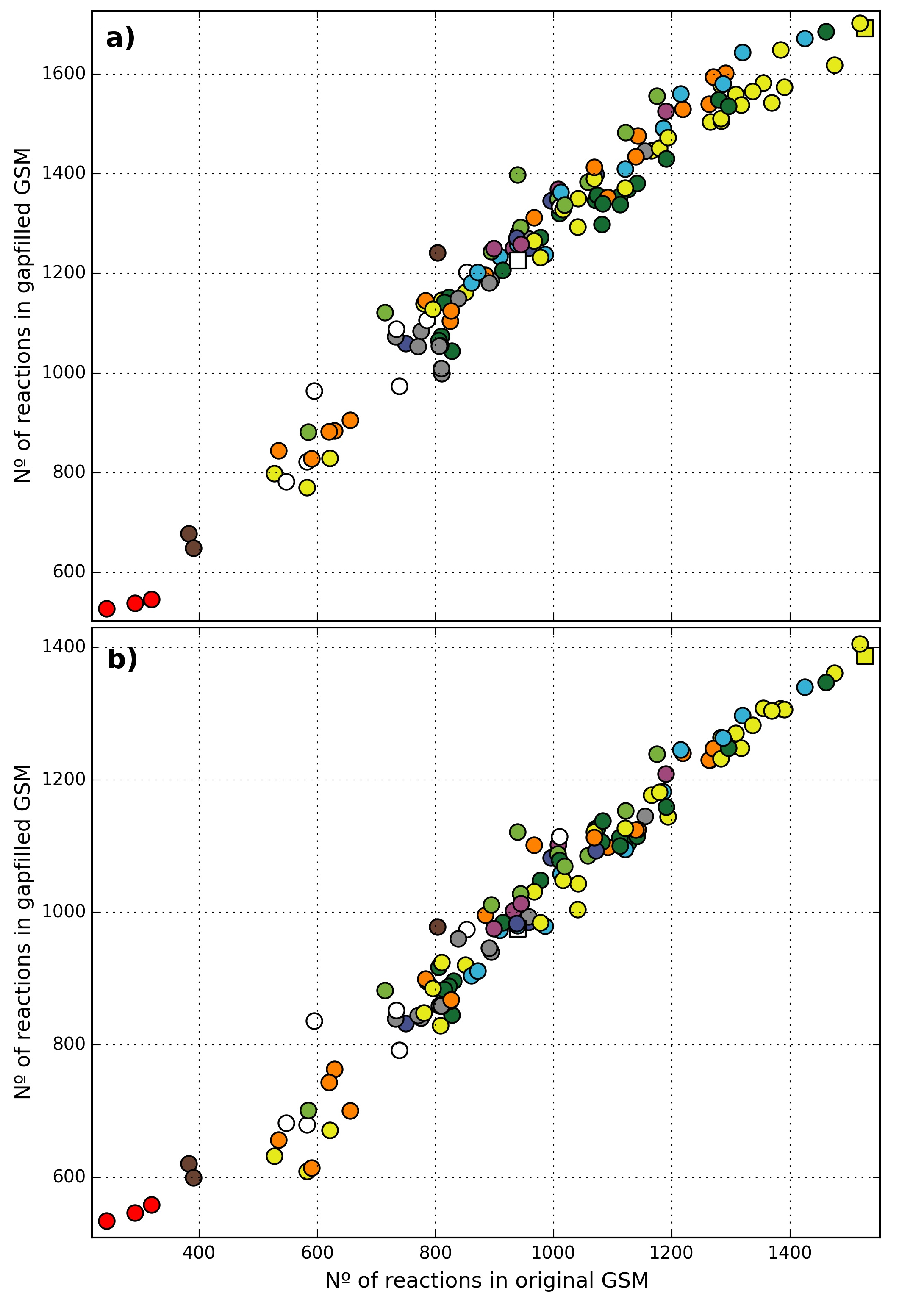

Supplement: S4 Fig — In a) and b) the number of reactions in the curated GSM against the number of reactions in the original GSM are shown (including metablocked reactions), using MM130.0 and MM130.1 respectively. (PNG) [file pone.0143626.s004.png]
